# Cigarette Smoking and Root Filled Teeth Extraction: Systematic Review and Meta-Analysis

**DOI:** 10.3390/jcm9103179

**Published:** 2020-09-30

**Authors:** Daniel Cabanillas-Balsera, Juan J. Segura-Egea, María C. Jiménez-Sánchez, Victoria Areal-Quecuty, Benito Sánchez-Domínguez, Paloma Montero-Miralles, Juan J. Saúco-Márquez, Jenifer Martín-González

**Affiliations:** 1 Department of Stomatology, Section of Endodontics, School of Dentistry, University of Sevilla, 41009 Sevilla, Spain; danielcaba@gmail.com (D.C.-B.); v.arealquecuty@hotmail.com (V.A.-Q.); beni2506@yahoo.es (B.S.-D.); montero_paloma@hotmail.com (P.M.-M.); jjsauco@us.es (J.J.S.-M.); 2 Materials Science Institute of Sevilla (ICMS), Joint CSIC-University of Sevilla Center, 41092 Sevilla, Spain; jimenezsanchez6@gmail.com

**Keywords:** endodontic medicine, endodontics, root canal treatment outcome, smoking habits, root-filled teeth extraction, tobacco smoking

## Abstract

Aim: The aim of this systematic review and meta-analysis was to investigate the possible association between smoking habits and the occurrence of root-filled teeth (RFT) extraction. Material and Methods: The Population, Intervention, Comparison, and Outcome (PICO) question was in adult patients who had RFT, does the absence or presence of smoking habits affect the prevalence of extracted RFT? Systematic MEDLINE/PubMed, Wiley Online Database, Web of Science, and PRISMA protocol was used to evaluate and present the results. The Grading of Recommendations, Assessment, Development, and Evaluation (GRADE) system was used for certainty in the evidence. The risk of bias was assessed according to Cochrane Collaboration common scheme for bias and ROBINS-I tool. Cumulative meta-analysis was performed with a random effects model. PROSPERO registration code: CRD42020165279. Results: After search strategy, 571 articles were recovered, seven were selected for full-text analysis, and two reported data on inclusion criteria, including 516 RFT, 351 in non-smokers, and 165 in smoker subjects. The meta-analysis provided an odds ratio indicating significant association between smoking and the prevalence of extracted RFT (OR = 3.43, 95% CI = 1.17–10.05, *p* = 0.02, I² = 64%). The certainty of the literature assessment was low per GRADE. Both studies were considered as moderate risk of bias. Conclusions: Tobacco smoking should be considered a negative prognostic factor for the outcome of root canal treatment, although the quality of the evidence is low. RFT of smoking patients are three times more likely to be extracted. Continuing to smoke after endodontic treatment may increase the risk of treatment failure. However, the overall strength of evidence is low. This must be considered a limitation of the present study and the conclusion should be valued with caution.

## 1. Introduction

Periapical inflammatory reaction caused and maintained by bacterial antigens from the root canal is named apical periodontitis [1]. Apical periodontitis (AP) is a very prevalent disease, ranging 0.6–20% of teeth [2,3]. The diagnosis of AP is made by analyzing the patient’s symptoms and signs, and can be confirmed radiographically by the disruption of the lamina dura and the presence of a radiolucent area encircling root apex, namely radiolucent periapical lesion (RPL) [4]. To cure AP, it is necessary to interrupt the passage of antigens from inside the root canal to the periapical tissue. This is achieved through root canal treatment (RCT) [5]. RCT can fail for different reasons [6], such as inadequate endodontic technique (including deficient aseptic control, missed canals, inadequate instrumentation, etc.) [7,8], root resorption, root fractures, or high bone lost [9,10,11], persisting periapical inflammation [12]. When it is not possible to perform non-surgical retreatment or apical surgery, RFT should be removed [13,14].

As mentioned above, the causes involved in extraction of RFT are many, being difficult to assess the role that each of them plays. One of the factors that has been studied in recent years is the systemic state of the patient [15,16]. Some systemic diseases can induce a pro-inflammatory status, altering immune response and impairing periapical healing [15,17]. This is the case of diabetes, which has recently been identified as a risk factor for non-retention of RFT [18].

Smoking coincides with diabetes in causing a systemic pro-inflammatory state and impaired immune response, having been associated with oral pathologies such as severe periodontal disease, pre-malignant lesions of the oral mucosa, oral cancer, caries, and high rate of treatment failure [19,20]. The dental pulp and periapical tissues of smokers show diminished defensive and reparative responses [21], as well as impaired bone healing [22]. Therefore, it is expected that the prevalence of AP is higher in smokers and, subsequently, that they also have a higher prevalence of RFT. A recently published systematic review with meta-analysis has concluded that smokers are 2.5 times more likely to have AP, being RCT almost three times more prevalent in smokers, compared to non-smoker subjects [23]. Moreover, a dose-response relationship between tobacco smoking and RCT have been found [24]. However, the possible effect of smoking on the outcome of endodontic treatment and its influence on RFT loss is uncertain, reaching several epidemiological studies contradictory conclusions [15,25,26]. The aim of this systematic review and meta-analysis was to investigate the possible association between smoking habits and the prevalence of extracted RFT.

## 2. Materials and Methods

The protocol of this systematic review has been developed and registered in the PROSPERO database (PROSPERO 2020 CRD42020165279). The systematic review has been developed according to the Preferred Reporting Items for Systematic Reviews and Meta-Analyses (PRISMA) Guidelines [27].

### 2.1. Review Question

The present review focused on the following research question: Does the presence or absence of smoking habits affect the prevalence of extracted RFT in adult patients? PICO (Population, Intervention, Comparison, and Outcome) schema for all the included studies to elaborate upon this research question were used to establish the eligibility criteria as follows:

Population: Adults patients with root-filled teeth.
Intervention: Presence of smoking habits, smoker.
Comparison: Absence of smoking habits, non-smoker.
Outcome: Extraction of root-filled teeth.

### 2.2. Inclusion and Exclusion Criteria

The inclusion criteria established were (a) epidemiological studies published between January 1980 to June 2020; (b) studies comparing smoking patients with non-smoking subjects; (c) studies including RFT; (d) studies providing data on the prevalence of extracted RFT, both in smoker subjects and in control non-smoking patients. Exclusion criteria were defined as (a) studies carried out in animals or in cell culture, and (b) studies reporting data only from smoking subjects. When there was no initial agreement among the reviewers, consensus was reached through dialogue.

### 2.3. Literature Search

Once the PICO question was established, the search strategy was designed [28,29]. Studies located in the search were selected according to inclusion and exclusion criteria, quality evaluation, and data extraction, and analysis. A literature search in MEDLINE/PubMed, Scopus, Web of Science, and Wiley Online Database was achieved using the following Mesh terms and keywords (Box 1): (Tobacco OR Smoking OR Smoker) AND (endodontic OR endodontics OR endodontic treatment OR root canal preparation OR root canal therapy OR root filled teeth OR endodontically treated teeth) AND (extraction OR retention OR dental avulsion OR avulsion OR tooth loss OR survival OR success OR failure OR outcome).

Box 1 **MeSH and key words combinations used for the search strategy for the Population, Intervention, Comparison, and Outcome (PICO) question: In adult patients who had root filled teeth, does the absence or presence of smoking habits affect the prevalence of extracted root filled teeth?**

((“tobacco”[MeSH Terms] OR “tobacco”[All Fields] OR “tobacco products”[MeSH Terms] OR (“tobacco”[All Fields] AND “products”[All Fields]) OR “tobacco products”[All Fields]) OR (“smoking”[MeSH Terms] OR “smoking”[All Fields]) OR (“smokers”[MeSH Terms] OR “smokers”[All Fields] OR “smoker”[All Fields])) AND (endodontic[All Fields] OR (“endodontics”[MeSH Terms] OR “endodontics”[All Fields]) OR (endodontic[All Fields] AND (“therapy”[Subheading] OR “therapy”[All Fields] OR “treatment”[All Fields] OR “therapeutics”[MeSH Terms] OR “therapeutics”[All Fields])) OR (“root canal preparation”[MeSH Terms] OR (“root”[All Fields] AND “canal”[All Fields] AND “preparation”[All Fields]) OR “root canal preparation”[All Fields]) OR (“root canal therapy”[MeSH Terms] OR (“root”[All Fields] AND “canal”[All Fields] AND “therapy”[All Fields]) OR “root canal therapy”[All Fields]) OR ((“plant roots”[MeSH Terms] OR (“plant”[All Fields] AND “roots”[All Fields]) OR “plant roots”[All Fields] OR “root”[All Fields]) AND filled[All Fields] AND (“tooth”[MeSH Terms] OR “tooth”[All Fields] OR “teeth”[All Fields])) OR (“tooth, nonvital”[MeSH Terms] OR (“tooth”[All Fields] AND “nonvital”[All Fields]) OR “nonvital tooth”[All Fields] OR (“endodontically”[All Fields] AND “treated”[All Fields] AND “teeth”[All Fields]) OR “endodontically treated teeth”[All Fields])) AND (extraction[All Fields] OR (“retention, psychology”[MeSH Terms] OR (“retention”[All Fields] AND “psychology”[All Fields]) OR “psychology retention”[All Fields] OR “retention”[All Fields]) OR ((“dental health services”[MeSH Terms] OR (“dental”[All Fields] AND “health”[All Fields] AND “services”[All Fields]) OR “dental health services”[All Fields] OR “dental”[All Fields]) AND (“fractures, avulsion”[MeSH Terms] OR (“fractures”[All Fields] AND “avulsion”[All Fields]) OR “avulsion fractures”[All Fields] OR “avulsion”[All Fields])) OR (“fractures, avulsion”[MeSH Terms] OR (“fractures”[All Fields] AND “avulsion”[All Fields]) OR “avulsion fractures”[All Fields] OR “avulsion”[All Fields]) OR (“tooth loss”[MeSH Terms] OR (“tooth”[All Fields] AND “loss”[All Fields]) OR “tooth loss”[All Fields]) OR (“mortality”[Subheading] OR “mortality”[All Fields] OR “survival”[All Fields] OR “survival”[MeSH Terms]) OR success[All Fields] OR failure[All Fields] OR outcome[All Fields])

A hand-search was also carried out in main endodontic journals (International Endodontic Journal, Journal of Endodontic, and Australian Endodontic Journal) and in the references of significant papers and reviews. The last search was made in June of 2020. 

Electronic and manual searches provided the titles and abstracts of articles related to the aims of the studies, which were categorized by three independent researchers (D.C.-B., J.M.-G., and J.J.S.-E.) according to the inclusion and exclusion criteria. Articles selected were full-text reviewed by four investigators (D.C.-B., J.M.-G., P.M.-M., and J.J.S.-E.).

### 2.4. Data Extraction

The methodology of selected studies was examined, and main features were extracted and compiled including, authors, date of publication, study design, subjects and sample size, main quantitative results and odds ratio values, and diagnosis of RPLs. Data extraction was performed by four investigators (D.C-B., J.M-G., M.C.J-S., and J.J.S-E). Disagreements were resolved by discussing between the four and reaching an agreement by majority. When necessary to clarify the data, the authors of the included studies were consulted.

### 2.5. Outcome Variables and Statistical Analysis

The primary outcome was the prevalence of extracted RFT. Odds ratio (OR), with its 95% confidence interval (CI) was calculated in every selected study trying to measure the effect of the relationship between smoking habits and the outcome of RCT. A random-effect model meta-analysis, on the basis of inverse variance method, was performed to determine the pooled OR and its 95% CI.

To estimate the variance and heterogeneity amongst trials, the Tau^2^ and the Higgins I^2^ tests were employed, taking into account that substantial heterogeneity is considered if I^2^ test is higher than 50% [30]. A funnel plot was plotted to illustrate the possible existence of publication bias [31]. To show the OR results, a forest plot [32] was used, along with the inverse variance pooled estimate. Finally, a level of *p* = 0.05 was considered significant. The meta-analyses were calculated with the 5.4 RevMan software (Review Manager Web. The Cochrane Collaboration, 2019. Available at revman.cochrane.org) [33].

### 2.6. Quality Evidence Assessment and Risk of Bias in Individual Studies

The quality of evidence of the included studies was analysed according to the guidelines provided by the Centre for Evidence-Based Medicine at Oxford [34]. The certainty in the evidence was assessed using the GRADE tool (GRADEpro GDT: GRADEpro Guideline Development Tool [Software]. Available from gradepro.org: https://gdt.gradepro.org/app/handbook/handbook.html#h.rkkjpmwb6m6z [35]. The GRADE tool has five domains: risk of bias, inconsistency, imprecision, indirectness, and publication bias, that can be downgraded and reduce the quality of the evidence [36]. Articles were assessed independently by 4 reviewers (J.J.S.E., J.M.G., D.C.B., and M.C.J.S.) and cases of disagreements in the risk of bias were discussed until a consensus was achieved.

The risk of bias of the included studies was assessed according to Cochrane Collaboration common scheme for bias and ROBINS-I tool [37], initially described to assess nonrandomized studies of interventions, but currently also available for observational designs (https://methods.cochrane.org/robins-i-tool).

## 3. Results

**Figure 1:**
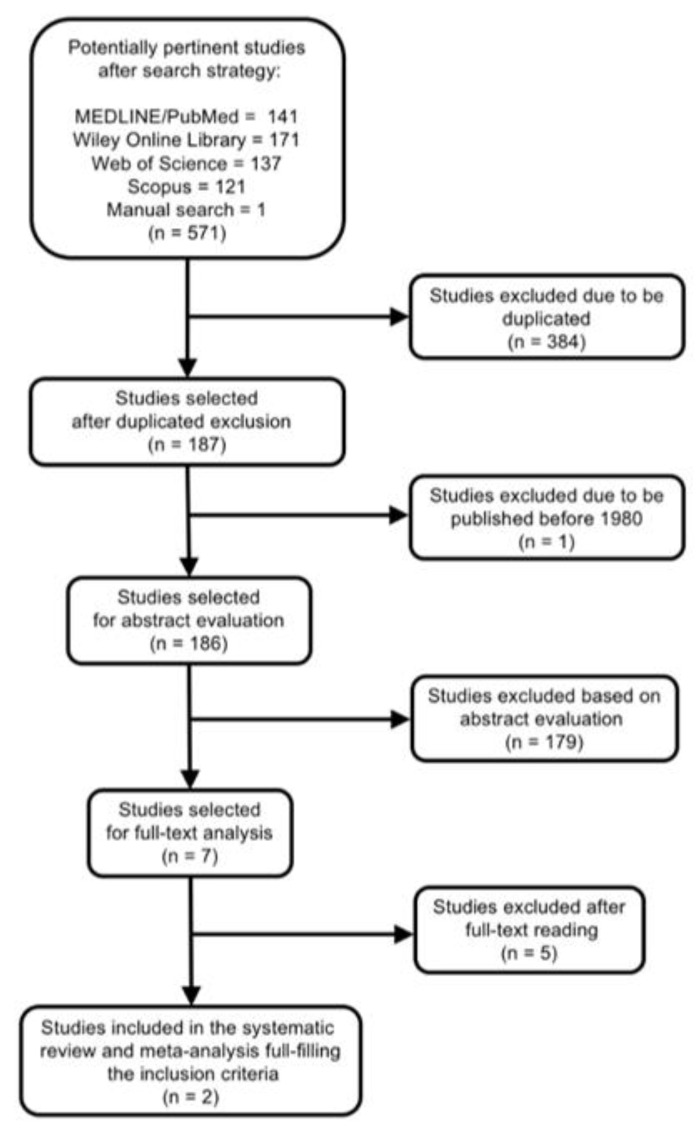
Flow diagram showing the process by which the studies about tobacco smoking and the prevalence of root filled teeth extraction were selected.

The search strategy is presented in Figure 1. After searching databases and hand-searching relevant bibliographies/papers, 571 articles were recovered. Excluding duplicates articles (*n* = 384) and publications before 1980 (*n* = 1), 186 articles were checked to satisfy the selection criteria by titles and abstract, declaring seven articles for full text review. Applying inclusion and exclusion criteria and assessing the level of evidence and the quality of all full articles read, only two were included in the meta-analysis. Five articles were excluded because of two reasons (Table 1): absence of data about how many RFT existed at the beginning of the study [38,39,40,41], and absence of data about how many RFT were extracted [17].

### 3.1. Study Characteristics

Finally, two studies were included in the analysis: 1. Doyle et al. [20], and 2. Khalighinejad et al. [42]. Study design, smokers and controls subjects, main data, and evidence level [34] are summarized in Table 2.

### 3.2. Meta-Analysis

A table of evidence was elaborated with data of both selected articles (Table 3). The estimated variance among the two studies was examined by Tau^2^ test, resulting non-significant (Tau^2^ = 0.41; Chi^2^ = 2.81; df = 1; *p* = 0.09).

**Figure 2:**
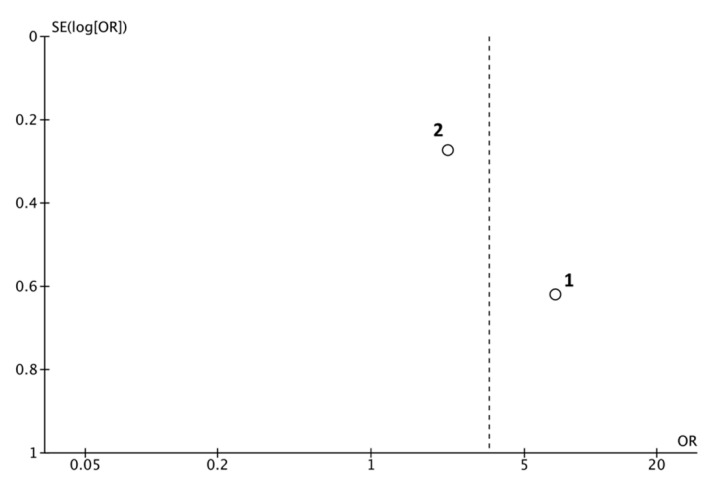
Funnel plot for estimates in meta-analysis of extracted root-filled teeth (RFT) in smoker subjects. Studies with higher power and lower standard error are placed towards the top. Studies with lower power are placed towards the bottom. 1. Doyle et al. (Scott L. Doyle et al., [20]) and 2. Khalighinejad et al. (Khalighinejad et al., [42]).

Heterogeneity test value (I² = 64%) was high; therefore, both weights were calculated using the random effects model, considering there was variation among the included studies and allowing the study outcomes to vary in a normal distribution (Figure 2, Funnel plot).

**Figure 3:**
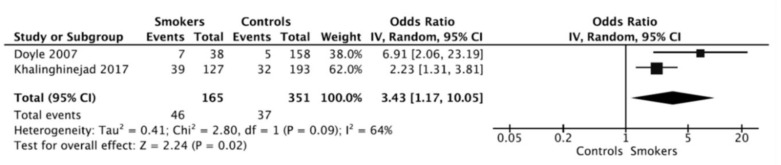
Forest plot of ORs and 95% confidence limits (CL) for the comparison of smokers and healthy control subjects regarding the frequency of extracted root-filled teeth (RFT). Overall estimate is based on data from the two studies. Black squares represent the point estimate of the odds ratio and have areas proportional to study size. Lines represent 95% confidence intervals. The diamond shows the summary statistic for the two studies. The solid line indicates an odds ratio of 1.0, and the dashed line indicates the overall odds ratio. OR: odds ratio; LCL: lower confidence level; UCL: upper confidence level.

Overall OR was calculated using inverse variance method with random effects, resulting an OR = 3.43 (95% CI = 1.17–10.05; *p* = 0.02). The ORs for each study and the pooled OR from the meta-analysis were shows in a forest plot (Figure 3). This result indicates that there is a significant difference in the prevalence of extracted RFT between smoking patients and control subjects.

### 3.3. Interpretation and Assessment of the Included Studies

The two studies included in the meta-analysis are retrospective, being published in 2007 [43] and 2017 [42]. With the results of the two studies, data from 516 RFT, 351 non-smokers, and 165 smokers were collected. In the study of Doyle et al. [43], the information from 367 patient’s charts was collected for a period of time of 10 years and clinical data, number of RFT, periapical index of RFT, patient’s habits, and type of restorations were determined. A positive association between smoking habits and the extraction of RFT was found. Smokers showed significantly higher percentage of extracted RFT (18.4%) compared with non-smokers (3.2%) (OR = 6.91; 95% CI = 2.06–23.19; *p* = 0.00043). 

The second study, Khalighinejad et al. [42], classified RFT according to their periodontal status and in addition also collected personal data. Nine years after, the authors evaluated teeth that required extraction and the factors that were related to this. The outcome revealed an increased risk of RFT extraction between smokers (30.7%), compared with non-smokers (16.6%) (OR = 2.23; 95% CI = 1.31–3.81; *p* = 0.0029).

### 3.4. Quality Evidence Assessment

**Figure 4:**
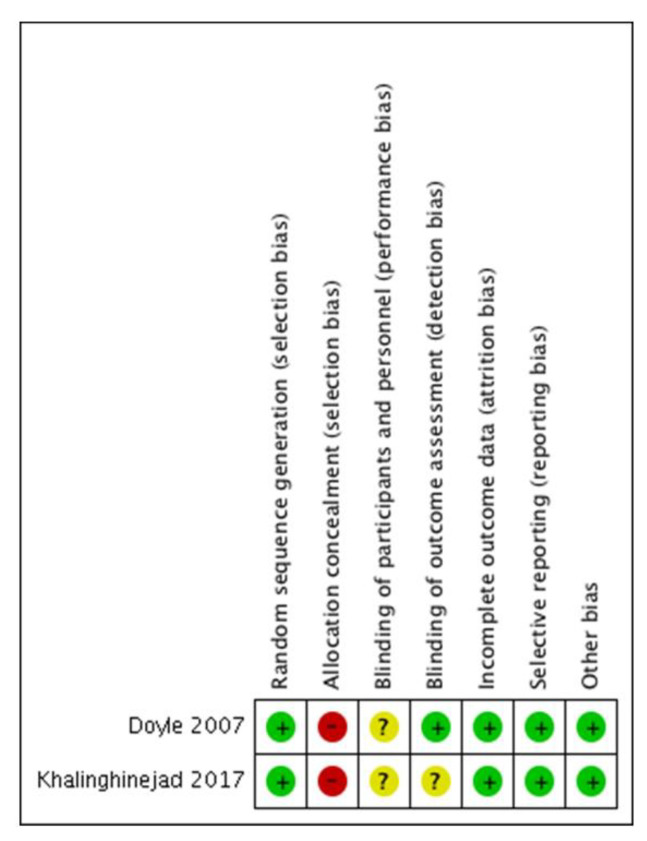
Risk of bias summary of included studies according the Cochrane Collaboration’s tool for assessing risk of bias. + Low risk of bias. - High risk of bias. ? Unclear risk of bias.

The scores [34] for the two studies were low, as both studies were scored with 3b (Table 2). The GRADE tool also demonstrated a low quality of the evidence for the included studies indicating that the true effect might be markedly different from the estimated effect (Table 4). Both included studies received the “serious” classification for the risk of bias, attending the limitations in the studies design and execution (see Figure 4—summary bias individual). Additionally, as detailed previously, the studies might have had substantial inconsistency, with an I^2^ statistic = 64% and Tau^2^ = 0.41, so the “serious” for the inconsistency factor was received. The “not serious” classification was assigned for the indirectness and imprecision domains, and as other considerations, a strong association was evidenced, and a plausible residual confusion would reduce proved effect.

According to ROBINS-I tool, both studies were considered as moderate risk of bias: Doyle et al. [20] with one domain classified as high and other as unclear, and Khalighinejad et al [42] with two unclear with other high risk of bias (Figure 4).

## 4. Discussion

This study aimed to analyze the possible link between smoking habits and the prevalence of extracted RFT. The results of the systematic review and meta-analysis carried out, including the available evidence about the prevalence of non-retained RFT, conclude that smoking is associated to RFT extraction.

After the literature search two studies providing follow-up data about RFT extraction were included: Doyle et al. [43] and Khalinghinejad et al. [42]. Both longitudinal studies analyzed the outcome of RCT in smokers and non-smoker patients, evaluating if the RFT was extracted or planned for extraction [42,43].

The random effects model was used to calculate overall ORs allowing the study outcomes to vary in a normal distribution. Furthermore, the heterogeneity value was moderate (64%), showing the relatively great variability between the studies. The inverse variance method reported an overall OR = 3.43 (*p* = 0.02), indicating that smokers are 3.4 times more likely to lose RFT, compared to non-smoker subjects. Both included studies are longitudinal, allowing a temporal link to be established between cigarette smoking and losing endodontically treated teeth.

The implications that the results of this meta-analysis have in the daily dental clinic are very important. Moreover, translational medicine involves bringing the results of epidemiological studies into clinical practice. Dentists must know that tobacco smoking is a negative prognostic factor for the outcome of endodontic treatment. Smoking patients should also know that their prognosis for RCT has lower expectations of success. If, in addition to smoking, patients have other risk factors such as cardiovascular disease [44,45] or diabetes [18,46,47], smokers should know that their RFTs have very high likelihood to end up being lost.

The association between smoking and the prevalence of extracted RFT had not been investigated so far by meta-analysis. Thus, the result of the present study fill this knowledge gap. However, the quality level of the two included studies is low (3b), because of this, the results of the present systematic review and meta-analysis should be valued with caution. The GRADE tool demonstrated an overall low strength of evidence. This implies that true effect might be markedly different from the estimated effect. Although the sample size is high in both studies, there are important drawbacks in their design that lowers up to “serious” their classification for the risk of bias. This must be considered a limitation of this study.

The present systematic review has other limitations. One is the possible bias of the results by the reason of the tooth extractions. The loss of RFT is interpreted as RCT failure, but it could be due to other simultaneous cofactors, such as periodontal disease, trauma, or increasing the age of patients [48,49,50]. Only one of the studies took into consideration the periodontal status of RFT [42]. Smoking periodontal patients probably display higher prevalence of extractions, since smoking is associated to severe periodontitis [51]. Teeth with moderate or severe periodontitis could have even three times more risk to lost, especially if these teeth do not receive an adequate periodontal treatment or if the patient are a smoker [42,52]. On the other hand, the two included studies did not take into account the dose-response effect. Then, it is impossible to determine if the alleged relationship meets the dose-effect criterion for be considered causal. Therefore, prospective studies are needed that take into account the dose-effect factor evaluating the amount of tobacco smoked and the time during which you have smoked. Another limitation of the present study is that grey literature has not been analyzed.

The results of this systematic review indicate that tobacco influences the post-endodontic periapical healing process, increasing three times the probability that treatment will fail and that the tooth will need to be extracted. The effect of cigarette smoking on periapical tissues repair after endodontic treatment has biological plausibility [15]. Several biological mechanisms can be argued. Tobacco smoking can affect the healing process of the RFT hindering bone repair and maintaining destructive periapical bone processes [15,53,54]. In this way, periapical lesion in the smoker’s RFT could have a slower healing process, establishing persistent apical periodontitis, which would lead to tooth loss [15,55]. Cigarette smoking alters leukocytes, macrophages, and T-cell lymphocytes functions, decreasing antibodies levels [56], and increasing the levels of pro-inflammatory mediators (IL−6, TNF- α, C-reactive protein) [54,57,58,59]. Alterations in microcirculation, both morphological and functional, could also have an impact on periapical healing after endodontic treatment and cause the loss of the RFT. It is possible that the inflamed periapical tissues of smokers have an insufficient supply of nutrients and oxygen [15]. Cigarette smoking would increase the level of carboxyhemoglobin as well as oxidative stress, altering micro-vascularization and decreasing the supply of oxygen and nutrients to the repair periapical tissues [60]. Furthermore, smoking is associated to delay fibroblast migration to the wound area and fibroblast dysfunction [61]. It has been shown that cigarette smoking has local and direct pro-inflammatory effect on inflamed periapical tissues, with increased levels of products of lipid peroxidation, such as 8-iso-PGF (2a), and products of the LOX-pathway [62]. Lastly, cigarette smoking, stimulating osteoclastic cells and reducing angiogenesis, impairs bone healing and tissues reparative response [63,64].

## 5. Conclusions

The results of the available studies indicate a significant relationship between smoking and higher prevalence of non-retained RFT. Tobacco smoking should be considered a negative prognostic factor for the outcome of root canal treatment. Dentists should know that RFT are much more likely to be extracted in smokers, explaining to patients that maintaining smoking after undergoing endodontic treatment can increase the risk of treatment failure. However, the meta-analysis includes only two studies, both of low quality level and serious risk of bias. Therefore, the overall strength of evidence is low, and this must be considered a limitation of the present study. Because of this, the conclusion should be valued with caution. Better designed longitudinal studies are needed to accurately define the impact of smoking on the outcome of endodontic treatment.

## Figures and Tables

**Table 1:** Studies excluded in the systematic review of association between smoking habits and the prevalence of root-filled teeth extraction. Excluded reason, authors, and year of these studies.

Excluded Reason	Authors	Year
Not provide necessary data to meta-analysis (absence initial n° RFT)	1. Zadik et al.	2008 [38]
	2. Zhong et al.	2010 [39]
	3. Touré et al.	2011 [40]
	4. Olcay et al.	2018 [41]
Not provide necessary data to meta-analysis (absence n° RFT extracted)	5. Marending et al.	2005 [17]

**Table 2:** Studies about smoking habits and the prevalence of root filled teeth extraction included in the systematic review. Study design, subjects and sample size, main results, and evidence level [34].

Authors	Year	Study Design	RCT	Association Diab.-Extr.RFT	Evidence Level [34]
1. Doyle et al.	2007 [20]	Retrospective chart review (follow up 1 years)	Controls: 158 Smokers: 38	YES; *p* = 0.0004	3b
2. Khalinghinejad et al.	2017 [42]	Longitudinal (retrospective 9 years)	Controls: 193 Smokers: 127	YES; *p* = 0.003	3b

RCT: root canal treatment; RFT: root-filled teeth; Extracted*RFT: extracted root-filled teeth.

**Table 3:** Studies about smoking habits and the prevalence of root filled teeth extraction. Results extracted and compiled, descriptive statistics and odds ratios calculated.

Authors	Year	Number of RFT	Non-Smoker Controls		Smoker Patients		OR (95% C.I.)	*p*
			Extracted*RFT/Total RFT	Extracted*RFT (%)	Extracted*RFT/Total RFT	Extracted*RFT (%)		
Doyle et al.	2007 [20]	196	5/158	3.2%	7/38	18.4%	6.9 (2.1–23.2)	0.0004
Khalinghinejad et al.	2017 [42]	320	32/193	16.6%	39/127	30.7%	2.2 (1.31–3.81)	0.003
**OVERALL**		516	37/351	10.5%	46/165	27.9%	**3.43** (1.2–10.1) *	**0.002**

RFT: root-filled teeth. Extracted*RFT: extracted root filled teeth. * Inverse variance method: Chi² = 2.80; *p* = 0.002.

**Table 4:** GRADE Working Group grades of evidence: Smoking habits and the prevalence of root filled teeth extraction.

Certainty Assessment							Certainty	Importance
Number of Studies	Study Design	Risk of Bias	Inconsistency	Indirectness	Imprecision	Other Considerations		
Extracted root filled teeth								
2	Observational studies	serious ^a^	serious ^b^	not serious	not serious	strong association all plausible residual confounding would reduce the demonstrated effect	**⊕⊕**○○ Low	IMPORTANT

Explanations: a. Detailed in Figure 4: Risk of bias summary, b. I^2^ = 64% and Tau^2^ = 0.41, High certainty: The authors have a lot of confidence that the true effect is similar to the estimated effect, Moderate certainty: The authors believe that the true effect is probably close to the estimated effect, Low certainty: The true effect might be markedly different from the estimated effect, Very low certainty: The true effect is probably markedly different from the estimated effect.
